# Androgen Receptor Gene CAG Repeat Polymorphism Regulates the Metabolic Effects of Testosterone Replacement Therapy in Male Postsurgical Hypogonadotropic Hypogonadism

**DOI:** 10.1155/2013/816740

**Published:** 2013-12-12

**Authors:** Giacomo Tirabassi, Nicola delli Muti, Giovanni Corona, Mario Maggi, Giancarlo Balercia

**Affiliations:** ^1^Andrology Unit, Division of Endocrinology, Department of Clinical and Molecular Sciences, Umberto I Hospital, Polytechnic University of Marche, 60126 Ancona, Italy; ^2^Endocrinology Unit, Azienda Usl di Bologna, Maggiore-Bellaria Hospital, Bologna, Italy; ^3^Andrology Unit, Department of Clinical Physiopathology, University of Florence, Florence, Italy

## Abstract

*Aim*. To evaluate the independent role of androgen receptor (AR) gene CAG repeat polymorphism on metabolic effects of testosterone replacement therapy (TRT) in male postsurgical hypogonadotropic hypogonadism, a condition frequently associated with hypopituitarism and in which the TRT-related metabolic effects are combined with those deriving from concomitant administration of metabolically active pituitary-function replacement therapies. *Methods*. 15 men affected by postsurgical hypogonadotropic hypogonadism were evaluated before and after TRT. Cardiovascular risk factors (CVRFs), pituitary-dependent hormones, and AR gene CAG repeat polymorphism were considered. *Results*. Testosterone, insulin-like growth factor 1 (IGF-1), and estradiol were the only hormones, which varied significantly between the two phases. All CVRFs significantly improved after TRT. The number of CAG triplets was positively and significantly correlated with all the variations (Δ-) of CVRFs (except for a significant negative correlation with Δ-high-density lipoprotein); the opposite occurred between the latter and Δ-testosterone. No correlation between Δ-IGF-1 or estradiol and Δ-CVRFs was found. At multiple linear regression, after correction for Δ-testosterone, nearly all the associations between the number of CAG triplets and Δ-CVRFs were confirmed. *Conclusions*. In male postsurgical hypogonadotropic hypogonadism, shorter AR gene CAG tract length seems to yield greater metabolic improvement after TRT, independently of the effects of concomitant pituitary-function replacement therapies.

## 1. Introduction

The androgen receptor (AR) gene is located on X chromosome at q11-q12 and is composed of eight exons [1]. Exon 1 of the AR gene contains a polymorphic sequence of CAG repeat, which varies in number from 10 to 35 [2] and which encodes polyglutamine stretches of AR transactivation domain [3]. Evidence suggests that CAG number is inversely correlated to the transcriptional activity of AR [2]. In fact, subjects affected by Kennedy Syndrome present a number of CAG repeats greater than 40 together with decreased virilization, testicular atrophy, reduced sperm production, and infertility [4]. Similarly, other studies have shown that shorter CAG repeats are associated with prostate cancer [5], benign prostatic hypertrophy [6], prostate increase during androgen treatment [7], improved seminal parameters [8, 9], and improved mineral bone density [10].

However, great uncertainty exists in the literature regarding the association between AR gene CAG repeat polymorphism and metabolic profile. In fact, while some authors found a connection of the low number of CAG repeats with low high-density lipoprotein (HDL) cholesterol levels [8, 11] and with increased severity of coronary heart disease [12], others reported a relationship between the high number of CAG repeats and worse cardiovascular risk factors (CVRFs) [13]. These results are derived from transversal studies which did not examine hypogonadal patients. In particular, very little is known about the role AR gene CAG repeat polymorphism plays in influencing long-term metabolic effects of testosterone replacement therapy (TRT).

Given this premise, we decided to evaluate the independent effect of AR gene CAG repeat polymorphism on TRT-related metabolic changes, focusing on male postsurgical hypogonadotropic hypogonadism, a condition which is nearly always associated with hypopituitarism and requires the concomitant administration of metabolically active hormone replacement therapies, such as recombinant growth hormone (GH), levothyroxine, and cortisone [14–18].

## 2. Materials and Methods

### 2.1. Subjects

15 males treated in our clinic were retrospectively evaluated. Inclusion criteria were, (a) hypogonadotropic hypogonadism [19, 20] secondary to surgical removal of pituitary adenoma, (b) absence of hypophyseal adenoma hypersecretion before surgery, (c) absence of hypophyseal hormone deficit before surgery, (d) absence of hypogonadism before surgery, (e) presence of clinical follow-up data for the whole length of the study, and (f) absence of previous diseases able to condition metabolic parameters considered in this study (e.g., diabetes mellitus, dyslipidaemia, arterial hypertension, etc.).

### 2.2. Study Protocol

The studied subjects underwent clinical and biochemical evaluation at the beginning of TRT (time 0) and before the eighth undecanoate testosterone injection (74–84 weeks after the first testosterone injection) (recovery phase). 1000 mg of undecanoate testosterone was administered intramuscularly after 6 weeks before the first injection (loading dose), followed by similar injections every 10–14 weeks, depending on blood testosterone values and clinical symptoms [21]. All subjects presented both somatotropic and gonadotropic function deficits [19, 20, 22]; among them, four had also thyreotropic [23], two also corticotropic [24], and three both thyreotropic and corticotropic function deficits. All patients were treated with appropriate doses of glucocorticoid (cortisone acetate, 37.5–50 mg daily), somatotropic (recombinant human GH, 0.3–0.6 mg daily), and thyroid (levothyroxine, 75–100 *μ*g daily) replacement therapy, depending on the specific hormone deficit. Glucocorticoid and thyroid replacement therapy began in the hours/days immediately after surgery, depending on the presence and degree of the specific deficit. Somatotropic replacement therapy and TRT were started between 6 and 12 months after surgery; no subject was given GH replacement therapy before TRT. Duration of hypogonadism was calculated as the time between surgery and beginning of TRT (time 0).

This is a retrospective study and the data examined were part of the diagnostic work-up, except for genetic data which were collected as part of a research protocol. The study was performed according to the Declaration of Helsinki and approved by the institutional ethics committee. An informed consent was obtained from each individual.

### 2.3. Clinical Evaluation

Among the measured clinical parameters, weight, waist circumference, systolic (SBP), and diastolic blood pressure (DBP) were considered in the analysis. Weight, waist circumference, SBP, and DBP were collected according to standard methods previously described [25, 26].

### 2.4. Biochemical and Hormone Evaluation

Blood samples were taken in the morning. The following biochemical and hormone parameters were considered: glycaemia, glycated hemoglobin (HbA1c), total cholesterol, HDL cholesterol, triglycerides, basal insulinemia, follicle-stimulating hormone (FSH), luteinizing hormone (LH), total testosterone, estradiol, free T3 (FT3), free T4 (FT4), cortisol, insulin-like growth factor-1 (IGF-1), and prolactin.

Plasma glucose was measured by photometric determination using the hexokinase method. HbA1c was measured in whole blood using ion-exchange high performance liquid chromatography with the Bio-Rad Variant Haemoglobin Testing System (Bio-Rad Laboratories, Hercules, CA, USA). Total cholesterol, HDL cholesterol, and triglycerides were assayed enzymatically with a final Trinder reaction (ADVIA 2400 SIEMENS, Bayer Diagnostics, Tarrytown, NY, USA). All hormone assays were carried out using immunoassay commercial kits. As far as total testosterone is concerned, its assay was performed using ADVIA centaur XP immunoassay, SIEMENS Medical Solution Diagnostics (minimum detectable concentration: 0.1 ng/mL; intra- and interassay coefficients of variation were, respectively, 6.2% and 4.4% at a testosterone concentration of 0.95 ng/mL and 4.7% and 4.7% at 3.65 ng/mL). The normal reference ranges for the biochemical parameters studied were the following: plasma glucose, 70–99 mg/dL; HbA1c, <6.0%; total cholesterol, <200 mg/dL; HDL cholesterol, >40 mg/dL for males; triglycerides, <150 mg/dL; basal insulinemia, 4–23 *μ*U/mL; FSH, 1.7–6.9 IU/L; LH, 1.6–10.0 IU/L; total testosterone, 3–8.5 ng/mL; estradiol, 11–47 pg/mL; FT3, 2.3–4.2 pg/mL; FT4, 0.8–1.8 ng/dL; serum cortisol (8.00 am), 7–27.5 mcg/dL; IGF-1, 66–251 ng/mL for males between 40 and 50 years; 57–221 ng/mL for males between 50 and 60 years; 46–211 ng/mL for males between 60 and 70 years; prolactin, 2–15 ng/mL.

Insulin sensitivity was assessed by the homeostasis model assessment of insulin resistance (HOMA-IR) [25], which is calculated as follows: [fasting plasma glucose (mmol/L) × fasting insulin (mU/L)]/22.5. Low density lipoprotein (LDL) cholesterol was calculated according to the Friedewald et al. [27] formula: LDL cholesterol = total cholesterol − HDL cholesterol − triglycerides/5.0 (mg/dL).

### 2.5. Polymerase Chain Reaction (PCR) Amplification and Sequencing

AR gene CAG repeats genotyping was performed as previously described [28]. Briefly, genomic DNA was isolated from peripheral leukocytes. PCR assay was performed with 150 ng DNA; primers were used at final concentration of 10 mM. The hot-start technique was used to prevent nonspecific amplification. PCR products were purified using QIAquick PCR Purification Kit (Qiagen, Hilder, Germany) and approximately 30 ng of the purified products was submitted to a sequencing reaction with the BigDye Terminator v3.1 Cycle Sequencing Kit (ABI Prism; Applied Biosystem). The samples were sequenced on a CEQ 2000 XL sequencer (Beckman Coulter, Fullerton, CA, USA).

### 2.6. Statistical Analysis

Shapiro-Wilk's test was applied to verify the normal distribution of the continuous variables. Data are expressed as median (interquartile range) when not-normally distributed and as mean ± standard deviation when normally distributed. CVRFs and hormone variations between the two phases (Δ-) were calculated as the value present at the recovery phase minus the value present at time 0; statistical comparison between the two phases was made using Student's *t*-test for paired data or the Wilcoxon test depending, respectively, on normal or nonnormal data distribution. In order to study the effect of AR gene CAG polymorphism on metabolic parameters, independently from that of the administered hormones, a two-step approach was adopted. First, Pearson correlations of the various significant hormonal variations and number of CAG triplets with Δ-CVRFs were carried out. Then, multiple linear regression analysis was performed including as dependent variables the Δ-CVRFs, one at a time, and as independent ones the variables which, in the Pearson correlation, were significantly correlated with Δ-CVRFs. Significance was set at *P* < 0.05. Statistical analyses were performed using SPSS 16 package (SPSS Inc., Chicago, IL, USA).

## 3. Results

Table 1 presents general and hormone parameters of the studied subjects. Testosterone, IGF-1, and estradiol varied significantly between the two phases, while the other hormones did not change significantly (Table 1). Conversely, all metabolic variables improved significantly after TRT (Table 2).  Table 3 shows the Pearson correlations of statistically significant hormonal variations (Δ-) between the two phases, that is, testosterone, IGF-1, and estradiol, and of the number of CAG triplets with the Δ-CVRFs. The number of CAG triplets correlated positively and significantly with all the Δ-CVRFs (except for the significant and negative correlation with Δ-HDL cholesterol) (Table 3). On the contrary, Δ-testosterone correlated negatively and significantly with all Δ-CVRFs (except for the significant and positive correlation with Δ-HDL cholesterol), while Δ-IGF-1 and estradiol did not correlate significantly with any of the Δ-CVRFs (Table 3). Multiple linear regression analysis was carried out including as dependent variables the Δ-CVRFs and as covariates both the number of CAG triplets and Δ-testosterone. After adjusting for Δ-testosterone, CAG repeat length was still positively and significantly associated with Δ-weight, Δ-glycemia, Δ-HbA1c, Δ-triglycerides, Δ-HOMA-IR, Δ-SBP, Δ-DBP, and negatively with Δ-HDL cholesterol (Table 4); positive and almost significant (*P* = 0.05) association was also evident with Δ-LDL cholesterol (Table 4).

## 4. Discussion

In this work we evaluated the impact of AR gene CAG polymorphism length on metabolic effects of TRT, focusing on male postsurgical hypogonadotropic hypogonadism, a condition in which this aspect has not yet been studied. As already anticipated, postsurgical hypogonadotropic hypogonadism is a particular type of hypogonadism in which TRT-related metabolic changes combine with those deriving from the frequent concomitant administration of pituitary-function replacement hormones [14–16, 29, 30]. In fact, administration of replacement cortisol therapy in subjects affected by adrenocortical failure increases and normalizes glycaemia and blood pressure and favours weight recovery [14]. GH replacement therapy, instead, produces an improvement of the lipid profile and increases waist circumference, body mass index (BMI), and glycaemia in patients with GH deficiency [15]. Similarly, levothyroxine administration in hypothyroid patients induces a decrease in total and LDL cholesterol [16]. Moreover, TRT in hypogonadal males is able to influence metabolic parameters causing a reduction of fasting plasma glucose, HOMA-IR, triglycerides, waist circumference, and usually augmenting HDL cholesterol [19, 31–34]; also, estrogens, which increase after TRT due to aromatase-mediated conversion [35, 36], have been found to be positively associated with metabolic syndrome [37].

In our subjects we found that shorter length of AR gene CAG repeat polymorphism favours the improvement of many of the CVRFs after TRT independently from the metabolic effects of the pituitary-function replacement therapies. Our findings agree with a large study carried out on 1859 men aged 20–79 years showing that subject with CAG repeat length lower than 22 had lower levels of BMI, glycaemia, SBP, and percentage of hypertension [13]. Moreover, our data confirm the tendency, already highlighted in the only other study on the same topic, which included 66 men affected by hypogonadism of various aetiologies (i.e., primary, secondary, and late onset hypogonadism), reporting that the SBP and DBP decrease following TRT occurred mainly in subjects with shorter CAG repeat length [38]. It is also worth mentioning that, in their study, no influence of AR gene CAG repeat polymorphism on lipid profile improvement was evident [38]; probably the heterogeneity of the sample examined by those authors and the different statistical methods used could explain this discrepancy.

Nevertheless, we cannot avoid mentioning those studies which found a completely different association between AR gene CAG polymorphism and metabolic profile. In fact, Skjærpe et al. studying 172 men, aged 60–80 years, found that those with a CAG length below 21 had higher values of glycaemia, c-peptide, and HbA1c, as compared to those with CAG length over 21 [39]. In line with these findings, in 131 men undergoing coronary angiography, it has been reported that those with short AR had significant coronary artery disease more frequently than men with long AR [12]. Moreover, in 110 healthy men the number of AR gene CAG repeats was found to be positively correlated with HDL cholesterol levels and flow-mediated vasodilatation, a measure of endothelial function [40].

Interestingly, in our sample TRT led to an increase in HDL cholesterol. This finding is in agreement with a recent meta-analysis [41] and with the previously mentioned work by Zitzmann and Nieschlag [38] who reported a significant increase in HDL cholesterol from 42.4 ± 11.3 mg/dL to 50.1 ± 8.9 mg/dL (resp., mean and standard deviation). However, at variance with this, some evidence shows that TRT may also have the opposite effect on HDL cholesterol levels in hypogonadal men [42, 43]. Although a variety of factors, like duration of study, fat distribution, obesity, diet, age, alcohol intake, exercise, and smoking [38, 43], have been considered to explain these diverse results, in our opinion more attention should be given specifically to the length of CAG polymorphism which could represent the dominating factor able to condition the variations of HDL cholesterol following TRT.

## 5. Conclusions

In conclusion, our study suggests that, in men affected by postsurgical hypogonadotropic hypogonadism, the shorter length of AR gene CAG tract is associated with an improved metabolic effect of TRT, independently of the effects of other concomitant pituitary-function replacement therapies. If our data will be confirmed by further studies, genetic analysis of AR gene CAG repeat polymorphism could be introduced in clinical practice as an adjunctive factor in evaluating cardiovascular risk in hypogonadal men undergoing TRT.

## Figures and Tables

**Table 1:** General and hormonal characteristics of studied subjects.

General characteristics				
Age (years)	55.66 ± 8.64			
Duration of hypogonadism (months)	8.46 ± 1.88			
Number of AR gene CAG triplets	18.13 ± 3.41			

Hormonal characteristics				
	Time 0	Recovery phase	Δ	*P* ^+++^

FSH (IU/L)	0.90 (0.70–1.10)	0.70 (0.50–0.90)	−0.40 (−0.60–0.20)	NS
LH (IU/L)	0.92 ± 0.40	0.78 ± 0.26	−0.14 ± 0.37	NS
Total testosterone (ng/mL)	1.53 ± 0.52	3.96 ± 0.36	2.42 ± 0.70	<0.001
Estradiol (pg/mL)	7.66 ± 1.95	32.80 ± 10.31	25.13 ± 11.13	<0.001
FT3 (pg/mL)	3.24 ± 0.50	3.11 ± 0.46	−0.12 ± 0.74	NS
FT4 (ng/dL)	1.24 ± 0.28	1.33 ± 0.18	0.08 ± 0.40	NS
Cortisol (mcg/dL)	14.70 (12–16.80)	13.50 (11.40–17.20)	0.80 (−5–1.5)	NS
IGF-1 (ng/mL)	34.93 ± 7.59	153.13 ± 41.05	118.20 ± 43.73	<0.001
Prolactin (ng/mL)	6.82 ± 2.97	7.35 ± 3.17	0.52 ± 4.86	NS

Values are expressed as median (interquartile range) or mean ± standard deviation.

^+++^Statistical comparison between time 0 and recovery phase.

AR: androgen receptor; Δ: variation; FSH: follicle-stimulating hormone; LH: luteinizing hormone; FT3: free T3; FT4: free T4; IGF-1: insulin-like growth factor-1; NS: not significant.

**Table 2:** Metabolic profile of subjects studied in the two phases.

	Time 0	Recovery phase	Δ	*P* ^+++^
Weight (kg)	79.8 ± 10.4	77.8 ± 10.9	−1.9 ± 2.0	0.002
Waist (cm)	95.7 ± 10.3	93.7 ± 9.7	−2.0 ± 1.4	<0.001
Glycaemia (mg/dL)	113.5 ± 12.0	105.6 ± 7.4	−7.8 ± 6.8	<0.001
HbA1C (%)	6.0 ± 0.1	5.9 ± 0.2	−0.1 ± 0.1	<0.001
Total cholesterol (mg/dL)	254.8 ± 39.8	236.6 ± 30.0	−18.1 ± 14.3	<0.001
HDL cholesterol (mg/dL)	39.8 ± 10.5	45.0 ± 6.1	5.2 ± 7.4	0.017
LDL cholesterol (mg/dL)	181 (138–226.6)	149 (131.4–188.4)	−16.6 ((−36)–(−4))	0.001
Triglycerides (mg/dL)	169.4 ± 29.2	159.4 ± 28.3	−10.0 ± 7.10	<0.001
HOMA-IR	9.7 ± 3.2	6.4 ± 1.8	−3.2 ± 1.9	<0.001
SBP (mmHg)	130 (120–140)	120 (110–130)	−10 ((−20)–(−10))	0.002
DBP (mmHg)	89.6 ± 7.8	83.0 ± 5.6	−6.6 ± 6.4	<0.001

Values are expressed as median (interquartile range) or mean ± standard deviation.

^+++^Statistical comparison between time 0 and recovery phase.

Δ: variation; HbA1c: glycated hemoglobin; HDL: high-density lipoprotein; LDL: low-density lipoprotein; HOMA-IR: homeostasis model assessment of insulin resistance; SBP: systolic blood pressure; DBP: diastolic blood pressure.

**Table 3:** Pearson correlations of statistically significant hormonal variations between the two phases and number of AR gene CAG triplets with CVRFs variations.

	Δ-Testosterone	Δ-Estradiol	Δ-IGF-1	Number of AR gene CAG triplets
Δ-Weight	*r*: −0.617	NS	NS	*r*: 0.755
	*P*: 0.014			*P*: 0.001
Δ-Waist	*r*: −0.633	NS	NS	*r*: 0.685
	*P*: 0.011			*P*: 0.005
Δ-Glycaemia	*r*: −0.758	NS	NS	*r*: 0.938
	*P*: 0.001			*P* < 0.001
Δ-HbA1c	*r*: −0.715	NS	NS	*r*: 0.921
	*P*: 0.003			*P* < 0.001
Δ-Total cholesterol	*r*: −0.750	NS	NS	*r*: 0.726
	*P*: 0.001			*P*: 0.002
Δ-HDL cholesterol	*r*: 0.838	NS	NS	*r*: −0.958
	*P* < 0.001			*P* < 0.001
Δ-LDL cholesterol	*r*: −0.840	NS	NS	*r*: 0.856
	*P* < 0.001			*P* < 0.001
Δ-Triglycerides	*r*: −0.757	NS	NS	*r*: 0.921
	*P*: 0.001			*P* < 0.001
Δ-HOMA-IR	*r*: −0.902	NS	NS	*r*: 0.937
	*P* < 0.001			*P* < 0.001
Δ-SBP	*r*: −0.801	NS	NS	*r*: 0.935
	*P* < 0.001			*P* < 0.001
Δ-DBP	*r*: −0.828	NS	NS	*r*: 0.965
	*P* < 0.001			*P* < 0.001

AR: androgen receptor; CVRFs: cardiovascular risk factors; Δ: variation; IGF-1: insulin-like growth factor-1; HbA1C: glycated hemoglobin; HDL: high-density lipoprotein; LDL: low-density lipoprotein; HOMA-IR: homeostasis model assessment of insulin resistance; SBP: systolic blood pressure; DBP: diastolic blood pressure; NS: not significant.

**Table 4:** Influence of AR gene CAG repeat length on metabolic parameters.

	AR gene CAG repeat length	
	Unstandardized *β* coefficient (95% CI)^+++^	*P*
Δ-Weight (kg)	0.454 (0.023–0.885)	0.041
Δ-Waist (cm)	0.218 ((−0.125)–(0.561))	NS
Δ-Glycaemia (mg/dL)	1.970 (1.195–2.745)	<0.001
Δ-HbA1c (%)	0.038 (0.022–0.053)	<0.001
Δ-Total cholesterol (mg/dL)	1.414 ((−1.562)–(4.390))	NS
Δ-HDL cholesterol (mg/dL)	−1.817 ((−2.487)–(−1.147))	<0.001
Δ-LDL cholesterol (mg/dL)	2.845 ((−0.005)–(5.696))	0.050
Δ-Triglycerides (mg/dL)	1.927 (1.023–2.832)	<0.001
Δ-HOMA-IR	0.349 (0.177–0.520)	<0.001
Δ-SBP (mmHg)	2.453 (1.333–3.574)	<0.001
Δ-DBP (mmHg)	1.671 (1.134–2.209)	<0.001

+++: adjusted for Δ-testosterone; AR: androgen receptor; CI: confidence interval; Δ: variation; HbA1c: glycated hemoglobin; HDL: high-density lipoprotein; LDL: low-density lipoprotein; HOMA-IR: homeostasis model assessment of insulin resistance; SBP: systolic blood pressure; DBP: diastolic blood pressure; NS: not significant.
